# Acute effects of a single bout of high-intensity strength and endurance exercise on cognitive biomarkers in young adults and elderly men: a within-subjects crossover study

**DOI:** 10.1186/s12967-025-06685-y

**Published:** 2025-06-19

**Authors:** Carolin Haberstroh Bekkos, Md Abu Jafar Sujan, Astrid Kamilla Stunes, Atefe Rafiee Tari, Norun Aagård, Cathrine Langlie Brobakken, Martin Siksjø Brevig, Unni Syversen, Eivind Wang, Mats Peder Mosti

**Affiliations:** 1 Department of Research and Development, Clinic of Substance Use and Addiction Medicine, St. Olavs Hospital, Trondheim University Hospital, Trondheim, Norway; 2 Department of Mental Health, Faculty of Medicine and Health Sciences, Norwegian University of Science and Technology (NTNU), Trondheim, Norway; 3 Department of Circulation and Medical Imaging, Faculty of Medicine and Health Sciences, NTNU, Trondheim, Norway; 4 Department of Women’s Health, St. Olavs Hospital, Trondheim University Hospital, Trondheim, Norway; 5 Department of Clinical and Molecular Medicine, Faculty of Medicine and Health Sciences, NTNU, Trondheim, Norway; 6 Center for, Oral Health Services and Research, Mid-Norway (TkMidt), Trondheim, Norway; 7 Department of Neurology and Clinical Neurophysiology, St. Olavs Hospital, Trondheim University Hospital, Trondheim, Norway; 8 Myworkout, Medical Rehabilitation Clinic, Trondheim, Norway; 9 Department of Endocrinology, St. Olavs Hospital, Trondheim University Hospital, Trondheim, Norway; 10 Faculty of Health Sciences and Social Care, Molde University College, Molde, Norway; 11 Department of Psychosis and Rehabilitation, Psychiatry Clinic, St. Olavs Hospital, Trondheim University Hospital, Trondheim, Norway

**Keywords:** Exercise, Strength training, HIIT (high-intensity interval training), BDNF, Klotho, GPLD1, Cognition, Gene expression, Skeletal muscle, Serum

## Abstract

**Background:**

Although evidence for exercise-induced changes in neurocognitive biomarkers is emerging, research examining acute responses to different exercise regimes across sex and age is lacking. This study investigated serum concentrations of three neurocognitive biomarkers (i.e., Klotho, brain-derived neurotrophic factor (BDNF), and glycosylphosphatidylinositol-specific phospholipase D1 (GPLD1)) after acute strength and aerobic exercise, along with skeletal muscle gene expression.

**Methods:**

In a within-subjects crossover design, blood samples of 19 young women, 20 young men, and 14 elderly men were taken before, immediately, 3 h and 24 h after one bout of strength training (ST) and high-intensity interval training (HIIT). Muscle biopsies were taken from a subgroup (n = 22) before, 3 h and 24 h after ST and HIIT for gene expression analyses. Time changes and baseline levels, including the influence of sex and age, were analyzed using a multilevel model and Welch’s analysis of variance, respectively. Biomarker levels were adjusted for exercise-induced plasma volume changes.

**Results:**

Serum concentration of all biomarkers increased after ST and HIIT but were not affected by sex or age. While serum Klotho and BDNF levels peaked immediately after exercise in all groups, serum GPLD1 levels were highest at 3 h (young groups only). Age was a determining factor for baseline measures; young men had higher and lower resting serum Klotho and BDNF concentration, respectively, than elderly men. Muscle gene expression of Klotho increased after both exercise modes, and BDNF and GPLD1 expression was reduced within 24 h.

**Conclusions:**

Circulating levels of biomarkers linked to brain health can acutely be increased by one bout of ST or HIIT. This increase might be related to altered gene expression of these proteins in skeletal muscle. Ultimately, this could have beneficial implications for the management of mental and neurocognitive impairments.

**Supplementary Information:**

The online version contains supplementary material available at 10.1186/s12967-025-06685-y.

## Introduction

In recent years, exercise has emerged as a powerful stimulus for neurogenesis, neuroplasticity, and subsequent brain adaptations [1]. Cognitive abilities are particularly important for overall functioning in everyday life [2], and exercise could prevent cognitive decline, and even improve cognition and brain function [3–5]. These are promising findings, yet a better mechanistic understanding of exercise-induced adaptations is needed. This requires investigation of underlying physiological processes, one of which is the release of signaling substances, often referred to as “exerkines” [6, 7]. Specifically, acute exercise can induce transient changes in exerkine levels, which can eventually mediate neuroprotective effects and benefit brain function [7, 8]. Thus, studying changes in concentration of exercise-sensitive biomarkers can provide valuable insights into immediate effects on neural viability, and the potential for long-term changes in brain function with regular exercise [9, 10].

Two biomarkers that have gained growing attention in the past few years are soluble Klotho and glycosylphosphatidylinositol-specific phospholipase D1 (GPLD1). Klotho, which has a proposed role in (anti-)ageing, is a circulating factor that could potentially affect brain function positively [11]. For instance, higher serum levels of Klotho were associated with greater cortical brain structures and connectivity [12], and improved cognition [13]. These findings are encouraging, as both acute aerobic exercise [14] and strength training (ST) [15] led to increased circulating Klotho levels in healthy individuals. Certainly, more research is needed to investigate the possible influence of age and sex. The liver-derived enzyme GPLD1 was found to improve neurogenesis and cognition [16]. Although Horowitz et al. observed this association in mice, the authors also reported a possible link between GPLD1 levels and exercise in humans as active elderly individuals exhibited higher GPLD1 levels than their sedentary counterparts [16]. However, the potential role of Klotho and GPLD1 in the exercise-cognition interplay is still unclear. It remains to be elucidated whether circulating GPLD1 levels increase following acute exercise, and whether this may differ with age and sex. There are also a lack of studies exploring the expression of Klotho and GPLD1 in skeletal muscle following acute exercise.

A well-established biomarker in the context of neurocognition is brain-derived neurotrophic factor (BDNF), playing a major role in synaptic plasticity and neural development [17]. While low levels of BDNF may be linked to cognitive impairment [18–20], exercise-induced increases in circulating BDNF levels could contribute to improve neurocognition [10, 21]. In that regard, increased levels of circulating BDNF have been frequently reported after aerobic exercise [22–26], yet fewer studies have addressed the acute effect of ST, resulting in inconsistent results and a lack of studies in the elderly population [22, 27]. Likewise, whether skeletal muscle is a site of upregulated BDNF expression after acute exercise is a topic of debate. Both unaffected [28, 29], and increased [30, 31] BDNF mRNA expression have been observed in skeletal muscle in the following hours after exercise. Also, the majority of these studies included young male participants only, limiting the generalizability of the findings. 

Both ST and endurance exercise benefit physical health [32]. Strength training is recognized as a vital countermeasure for age- and lifestyle-related decline in muscular function and musculoskeletal health [33]. Importantly, an association between muscular strength and cognition has been established in individuals with psychiatric disorders [34, 35], and ST improved neuroplasticity and cognitive abilities in elderly individuals [36–38]. Aerobic high-intensity interval training (HIIT) is regarded an efficient intervention for improving maximal oxygen uptake (V̇O_2max_), and cardiovascular and metabolic health across different age groups and populations [39, 40], with the potential to also improve cognition [41–43]. Although ST and HIIT appear beneficial for neurocognitive adaptations, limited information is available regarding acute exercise responses of biomarkers relevant for such improvements. Most studies examining neurocognitive exerkines have been restricted to a single population (in regard to age or sex) and exercise mode. Thus, the purpose of this study was to examine time course kinetics of three highly relevant biomarkers related to neural development and brain function after two different training modes and analyze the possible influence of sex and age. Specifically, we primarily aimed to investigate the acute effect of a single session of lower extremity ST and treadmill HIIT in young women and men, and in elderly men on circulating levels of Klotho, BDNF, and GPLD1. A secondary aim was to analyze mRNA expression of these proteins in the skeletal muscle following ST and HIIT. 

## Methods

The present study is a secondary analysis of previously published data material on exercise-induced biomarker response, and we used banked serum for all analyses. Several of the methods used have been described before [44, 45].

### Study design and participants

This is a crossover within-subjects trial with three groups. Participants were recruited from September 2018 to February 2019, via posters in local gyms and at the Norwegian University of Science and Technology, Trondheim, Norway. Fifty-three individuals were included in the study with the following inclusion criteria: age ≥ 18 years, and absence of illness or chronic conditions that could prevent participants from performing the physical tests or affect blood samples (see Table 1 for participants’ characteristics). The Regional Committee for Medical Research Ethics, Norway, approved the study (REK2018/926), and all participants signed a consent form before assessments.

**Table 1:** Baseline characteristics of participants

	Young women (n = 19)	Young men (n = 20)	Elderly men (n = 14)
Age (years)^a^	25 ± 2.3*	25.5 ± 2.2	68.9 ± 3.7
Weight (kg)	65.6 ± 7.5	77.2 ± 10.3*	79.2 ± 10.4
Height (cm)	169.1 ± 5.4	181.4 ± 6.2	180.1 ± 6.7
BMI (kg $\cdot$ m^−2^)	23.0 ± 2.9	23.5 ± 2.5	24.4 ± 2.5
V̇O_2max_ (L $\cdot$ min^−1^)	3.30 ± 0.48*	4.80 ± 0.64*	3.35 ± 0.54
V̇O_2max_ (mL $\cdot$ kg^−1^ $\cdot$ min^−1^)	50.46 ± 6.28	62.65 ± 8.26	42.48 ± 5.35
HR_max_ (beats per min)^a^	195 ± 9	196 ± 7	169 ± 17
1RM (kg)^a,b^	104.2 ± 24.9	134.2 ± 28.8	130.4 ± 16.7

Data are presented as mean ± standard deviation. * = non-normally distributed data; *BMI* body mass index, *V̇O*_*2max*_ maximal oxygen uptake, *HR*_*max*_ maximal heart rate, *1RM* one repetition maximum. ^a,b^ n = 1 missing (a: young women, b: young men)

**Fig. 1:**
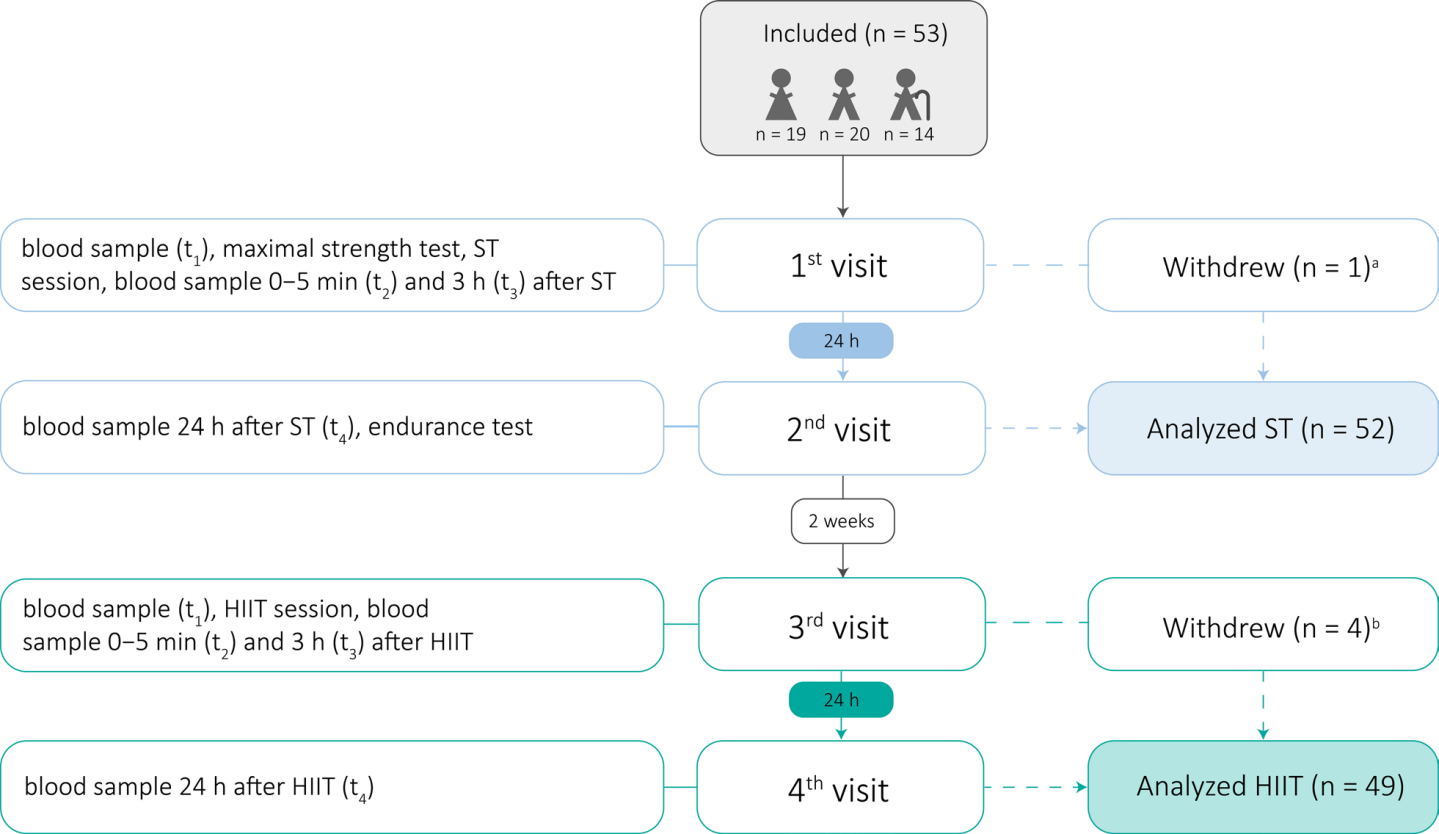
Flow chart. ^a^ 1 young woman withdrew, but participated in HIIT. ^b^ 2 young women, 1 young man, and 1 elderly man withdrew, but participated in ST. ST = strength training, HIIT = high-intensity interval training. Muscle biopsies were taken from a subgroup of volunteers at baseline (1st visit only), 3 h and 24 h after ST and HIIT (n = 22, 6 young women, 9 young men, 7 elderly men)

A study flow chart is presented in Fig. 1. Participants visited the laboratory at St. Olavs Hospital, Trondheim University Hospital, Trondheim, Norway a total of four times. All participants performed one acute bout of ST and HIIT, one after another with 2 weeks between sessions. All testing and training sessions were performed in a supervised laboratory setting, and the participants were requested to refrain from strenuous exercise 48 h before the testing/training sessions and in the 24-h period between blood tests.

### Assessments 

All testing and training sessions were carried out between 8:00 a.m. and 10:00 a.m. The participants completed a questionnaire about their medical history, lifestyle, and oral contraceptive use (young women). Anthropometric measurements were performed using a wall-mounted stadiometer and an electronic scale to assess height and weight, respectively, and body mass index (BMI, kg $\cdot$ m^−2^) was calculated. Furthermore, as specified below, all participants underwent blood sampling, performed two physical tests, and one bout of ST and HIIT (2 weeks apart). All participants were offered pure carbohydrate energy gel (60 mL; 87 kcal; Go Isotonic Energy, Science in Sport, London, UK) before the ST (one pack) and the HIIT (two packs) sessions. 

### Physical tests

#### One repetition maximum

Maximal strength was measured as one repetition maximum (1RM, which is the maximal weight an individual can lift once in one exercise) in a horizontal dynamic leg press (Technogym, Italy) following a protocol of the American College of Sports Medicine [46]. The 1RM was assessed after two warm-up sets with 5–8 repetitions each (one set at 40–60% and one set at 60–80% of estimated 1RM). To measure 1RM directly, we increased the weight by 4–8 kg after each successful attempt (2–3 min break in between trials) until the participant failed to lift the weight or the knee joint angle was not 90°. The mean 1RM values of the participants are presented in Table 1.

#### Maximal oxygen uptake

We assessed aerobic fitness level via measuring V̇O_2max_ directly with cardiopulmonary exercise testing on a treadmill (Woodway, Germany), using a metabolic gas analyzer (MetaMax Cortex II, Germany). Before the test started, participants warmed up for approximately 10 min. Then, 5.3% was used as the minimum inclination, and the speed was increased every 2–3 min until exhaustion. We identified V̇O_2max_ as the mean of the three highest values during the last min of the test. Maximal effort was identified on reaching at least one of the following criteria: plateau in oxygen uptake despite increasing workload; respiratory exchange ratio ≥ 1.05; and blood lactate concentration ≥ 8–9 mmol $\cdot$ L^−1^ [47, 48]. Maximal heart rate (HR_max_) was assessed with a Polar H10 heart rate sensor and a Polar M200 watch (Polar Electro, Finland) as the highest HR recorded during the test + three beats per min [49], which was used to determine the workload for the HIIT session. Participants’ mean values of V̇O_2max_ and HR_max_ are presented in Table 1.

### Exercise sessions

#### Strength training

After the 1RM test, participants performed a single ST session using the leg press, consisting of 4 sets with 8–10 repetitions with a load of 80% of 1RM. If the participant managed to perform 10 repetitions at 80% of 1RM easily, we increased the load by 4–8 kg in the next set. We instructed the participants to execute the exercise with a 90° angle and a slow eccentric and fast concentric movement. Rest for 2–3 min was included between each set of attempts.

#### High-intensity interval training

Participants performed one acute bout of HIIT on their 3rd visit. As described earlier [47], the session started with a 10-min warm-up at 60–70% of V̇O_2max_, followed by four 4-min intervals at 90–95% of HR_max_ at 5.3% of inclination. The participants were instructed to exercise at approximately 70% of HR_max_ for 3 min in between the high-intensity intervals (i.e., active break). To ensure target HR, participants used a Polar H10 and M200 watch, and were supervised by research staff.

### Blood sampling and serum analyses

Blood samples were collected by standard venipuncture at the following four time points: at baseline (i.e., before physical testing/exercise, pre, t_1_), immediately after (i.e., 0–5 min, post, t_2_), 3 h (t_3_) and 24 h (t_4_) after the acute exercise bouts. Baseline and 24 h blood samples were drawn after an overnight fast, but due to the participants ingesting carbohydrate energy gel before exercise sessions, blood sampling immediately and 3 h after exercise was performed non-fasting. However, the procedure was standardized for all participants as they consumed the same amount of gel and were only allowed to drink water. Blood samples (5 mL) were collected in vacuum tubes and centrifuged (3000 g/4 °C/10 min) after incubation at room temperature for 30 min. Subsequently, serum was aliquoted and stored at −80 °C until further analyses. The Department of Biochemistry, St. Olavs Hospital, Trondheim University Hospital, Trondheim, Norway analyzed total calcium levels for plasma volume assessments using accredited analysis procedures. Enzyme-linked Immunosorbent Assay (ELISA) kits were used to analyze biomarkers. Serum α-Klotho levels were analyzed using a human soluble α-Klotho (hereafter called Klotho) assay kit (Immuno-Biological Laboratories Co, Ltd., Naka Fujioka-Shi, Gunma, Japan; Cat# 27998; RRID:AB_2750859; detection limit 6.15 pg $\cdot$ mL^−1^). Serum BDNF levels were analyzed using a human free BDNF ELISA kit (Bio-Techne Ltd., Abingdon, UK; Cat# DBD00; RRID:AB_2924803; detection limit 20 pg $\cdot$ mL^−1^). Serum GPLD1 levels were analyzed using a human GPLD1 ELISA Kit (MyBioSource Inc., San Diego, CA, USA; Cat# MBS2021089; RRID:AB_3675887; detection limit 0.35 ng $\cdot$ mL^−1^). Intra- and inter-assay variations were 3% and 7% for the Klotho assay, 5% and 9% for the BDNF assay, and 10% and 12% for the GPLD1 assay, respectively. Analyses were performed in compliance with the manufacturers’ instructions.

### Correction for changes in plasma volume

The following procedure for plasma volume correction has previously been published [44]. Transient fluid shifts due to hemoconcentration and hemodilution can cause changes in plasma volume (∆PV) during and after exercise [50]. These changes can influence the interpretation of biomarker level measurements in the blood and should therefore be adjusted for [50, 51]. The equation by Dill and Costill [51], using hemoglobin and hematocrit concentrations, is considered the standard method to calculate %∆PV. The latter correlates well with changes in serum total calcium levels, which can be used as a biomarker for hemoconcentration [52]. In the present trial, serum analyses from t_2_–t_4_ were corrected for %∆PV using percentage change in calcium concentration ([Ca]) from the corresponding values at t_1_. The following equations were applied:

A) $${\%\Delta PV}_{tx}=100\cdot \left(\frac{{\left[Ca\right]}_{tx}- {\left[Ca\right]}_{t1}}{{\left[Ca\right]}_{t1}}\right)$$

B) $$\left[biomarker\right]{corrected}_{tx} = {\left[biomarker\right]}_{tx} \times \left(1- \left(\frac{{\%\Delta PV}_{tx}}{100}\right)\right)$$

where t_x_ represents the time point for measurements (t_2_–t_4_) after baseline (t_1_) in the respective training mode. All statistical analyses for biomarker serum levels at baseline and later time points were performed using %∆PV corrected values. Values for serum total calcium and percentage change of calcium for all time points can be found in the study by Stunes et al. [44].

### Biopsies

The procedure for obtaining muscle biopsies has been described previously [45]. Muscle biopsies were taken from a subgroup of participants who volunteered for this (n = 22), at baseline, and at 3 h and 24 h after each training session. To reduce discomfort, the baseline biopsy from the 1 st visit was used as a reference for the 3 h and 24 h biopsies after both ST and HIIT sessions. Two different types of needles (Bergstrøm, Pelomi, 6 mm, Albertslund, Denmark and Zamar vantage “B” type, Zanotti, 2 mm, Croatia) were used to obtain muscle tissue (200–300 mg) under local anesthesia (Xylocaine-adrenaline: 10 mg $\cdot$ Ml^−1^ lidocaine + 5 µg $\cdot$ mL^−1^ adrenalin). The dominant leg (preferred for kicking a ball) was chosen, and muscle biopsies were taken in the middle of m. *vastus lateralis*. Biopsies were collected 1 cm apart (proximally or distally from previous samples), 3.5 cm deep, and 15 cm proximal to the knee, close to the distal ventral midline of the muscle with no exemptions [53]. A bundle of muscle tissue was collected in cryo-tubes (after removing connective and fat tissue), immediately frozen in liquid nitrogen, and stored at −80 °C until further analyses.

### mRNA isolation and cDNA synthesis from muscle

As described previously [45], frozen muscle tissue ($\sim$ 30 mg) from the m. *vastus lateralis* was lysed in RNeasy lysis buffer with an electric knife-homogenizer. A RNeasy Fibrous Tissue Mini Kit (Qiagen) was applied to isolate total RNA according to the manufacturer’s instructions. Total RNA in each sample was then measured using a NanoDrop Spectrophotometer. Equal amounts of total RNA from each sample were applied directly to obtain a first-strand complementary DNA (cDNA), using the qScript cDNA Synhesis kit (Quantabio).

### Real-time PCR analyses

Real-time PCR (Polymerase Chain Reaction) analyses were performed with TaqMan^™^ Fast Advanced Master Mix (ThermoFisher) and QuantStudio^™^ 5 System (Thermo Fisher Scientific). Primers and probes were purchased from Thermo Fisher Scientific: glyceraldehyde-3-phosphate dehydrogenase (GAPDH), assay ID—Hs02786624_g1; Klotho, assay ID—Hs00934627_m1; BDNF, assay ID—Hs02718934_s1; GPLD1, assay ID—Hs00946499_m1. Relative mRNA expression of Klotho, BDNF, and GPLD1 was calculated applying the standard curve method, with GAPDH as reference/housekeeping gene.

### Statistical analyses

For analysis of serum levels at baseline and across time, we categorically coded the variables *group* with three levels (i.e., young women, young men, elderly men) and *time* with four levels (i.e., t_1_–t_4_). Importantly, we only considered differences between young women and young men, and between young men and elderly men to explore sex and age differences, respectively (i.e., differences between young women and elderly men are not reported). Additionally, as we chose to focus on serum level changes relative to baseline, differences between other time points (i.e., between t_2_, t_3_, and t_4_) are omitted. This applies also for skeletal muscle gene expression analyses. The two exercise modes (i.e., ST and HIIT) were analyzed separately.

For serum levels at baseline, data were tested for assumptions of one-way analysis of variance (ANOVA). Normal distribution was found to be acceptable using visual inspection of histograms and QQ plots. Three outliers (i.e., participants) each were excluded for Klotho and GPLD1 data (visual inspection of box plots). The homogeneity of variance assumption (analyzed using Levene’s test) was violated for all biomarkers, and thus, Welch’s ANOVA was performed. In case of significant overall differences in the variable *group*, Dunnett’s T3 post hoc test with adjustment for multiple testing was used to detect *group*-specific differences. Mean baseline values are reported as mean values from the 1 st and 3rd visit (Fig. 1). For participants with incomplete baseline measurements (data available from either the 1 st or 3rd visit only), the baseline value from the respective visit available was included (n = 7).

**Fig. 2:**
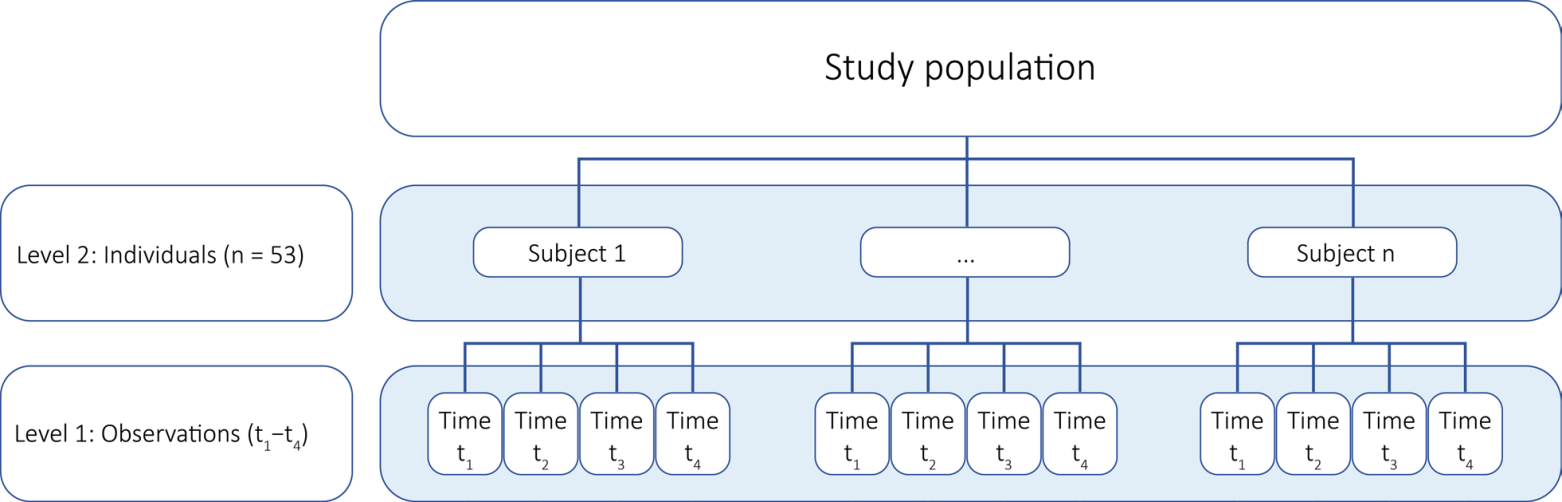
Structure of levels used in the analysis. Observations t_1_ = baseline (pre), t_2_ = immediately after exercise (post), t_3_ = 3 h after exercise, t_4_ = 24 h after exercise. Modified from Monsalves et al. [54]; http://creativecommons.org/licenses/by/4.0/

For analysis of our primary aim, we applied a multilevel model including two levels (Fig. 2) with the predictors *time, *group, and the interaction term time × group as fixed categorical factors. The random factor *subject* was added to allow for variation between individuals (see Supplementary Table 1 for coefficients of intraclass correlation). Restricted maximum likelihood was used as estimation method, and Satterthwaite approximation was applied to estimate degrees of freedom. More information on missing data, etc. can be found in the supplementary material. Assumptions as normally distributed residuals on levels 1 and 2 (analyzed graphically using histograms and QQ plots) and homoscedasticity of residuals on level 1 (analyzed graphically by residuals vs. predicted plot) were satisfied for all biomarkers except GPLD1 (heteroscedasticity, and non-normal distribution on level 2), and a natural log transformation was performed. In addition, one outlier each (i.e., participant) was excluded from the analysis of Klotho and GPLD1. Regarding collinearity assumption, our two explanatory variables time and group were not expected to be correlated. However, we formally tested collinearity using a chi-square test, which confirmed independence between the predictors.

For analysis of our secondary aim, we applied a multilevel model including two levels with the predictor *time* as fixed categorical factor, and a random intercept. Due to small sample size in each group (6 young women, 9 young men, 7 elderly men), we did not include group as fixed factor. While all assumptions (described above) were satisfied for Klotho, we excluded one outlier for GPLD1 data and naturally log transformed BDNF data since level 1 residuals were heteroscedastic.

In case of significant effects of *time* and/or *group*, pairwise comparisons using Šidák adjustment for multiple testing were performed. Effect sizes are presented as percentage change (significant t_1_ vs. t_2_ changes for primary aim only) and mean difference (MD) with 95% confidence intervals (CIs). Note that the difference between two log-transformed means is the log of the ratio and cannot be interpreted on its original scale as MDs or CIs. All presented MDs for log-transformed data were manually calculated from back-transformed estimated means at the specific time points. A ratio of means is presented instead of CIs. Estimates of variance components (i.e., between- and within-subject variability), along with 95% CIs, are provided in the supplementary material. All statistical analyses were performed using IBM SPSS Statistics version 29.0.1 (Armonk, NY, USA), and graphs were created using GraphPad Prism version 10.1.2 (Boston, MA, USA). Two-sided *p* < 0.05 were considered statistically significant.

## Results

### Baseline serum levels

F-statistic values from analysis of overall *group* differences for Klotho, BDNF and GPLD1 are presented in Table 3.

**Fig. 3:**
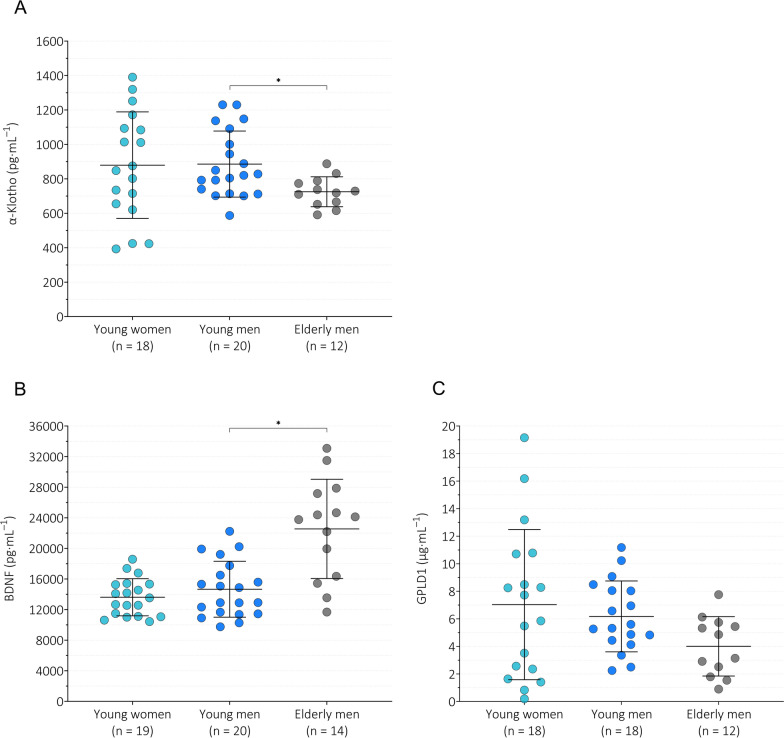
Baseline serum levels of α-Klotho (**A**), BDNF (**B**) and GPLD1 (**C**). *BDNF* brain-derived neurotrophic factor, *GPLD1* glycosylphosphatidylinositol specific phospholipase D1. Data are presented as means with error bars representing standard deviation. Welch’s ANOVA with Dunnett’s T3 post hoc test to adjust for multiple testing was used for detecting possible differences in sex (young women vs. young men) and age (young men vs. elderly men). *p < 0.01

For Klotho (Fig. 3A), a difference was observed between groups (*p* = 0.006), with higher serum Klotho levels in young men than in elderly men (MD = 160.59 pg $\cdot$ mL^−1^, 95% CI [34.84, 286.34], *p* = 0.009).

For BDNF (Fig. 3B), a difference was observed between groups (*p* < 0.001), with higher serum BDNF levels in elderly men than in young men (MD = 7892.97 pg $\cdot$ mL^−1^, 95% CI [2891.44, 12894.50],* p* = 0.002).

For GPLD1 (Fig. 3C), a difference was observed between groups (*p* = 0.029). However, the mean difference between young and elderly men (MD = 2.17 µg $\cdot$ mL^−1^, 95% CI [− 0.04, 4.38], *p* = 0.055) did not reach statistical significance after adjusting for multiple testing.

### Serum level changes across time

Overall, no statistically significant time × group interactions were found for any of the three biomarkers, for either ST or HIIT (Klotho ST: *p* = 0.206, HIIT: *p* = 0.140; BDNF ST: *p* = 0.059, HIIT: *p* = 0.287; GPLD1 ST: *p* = 0.356, HIIT: *p* = 0.326). F-statistic values from analysis of overall main effects, and interaction effects can be found in Table 3. The variance components estimates for Klotho and BDNF (variance, 95% CI, and SD) for ST and HIIT are presented in Supplementary Table 2. Since log-transformed estimates for variance components cannot be back-transformed to the original scale, they are omitted for GPLD1 data.

#### Klotho

**Fig. 4:**
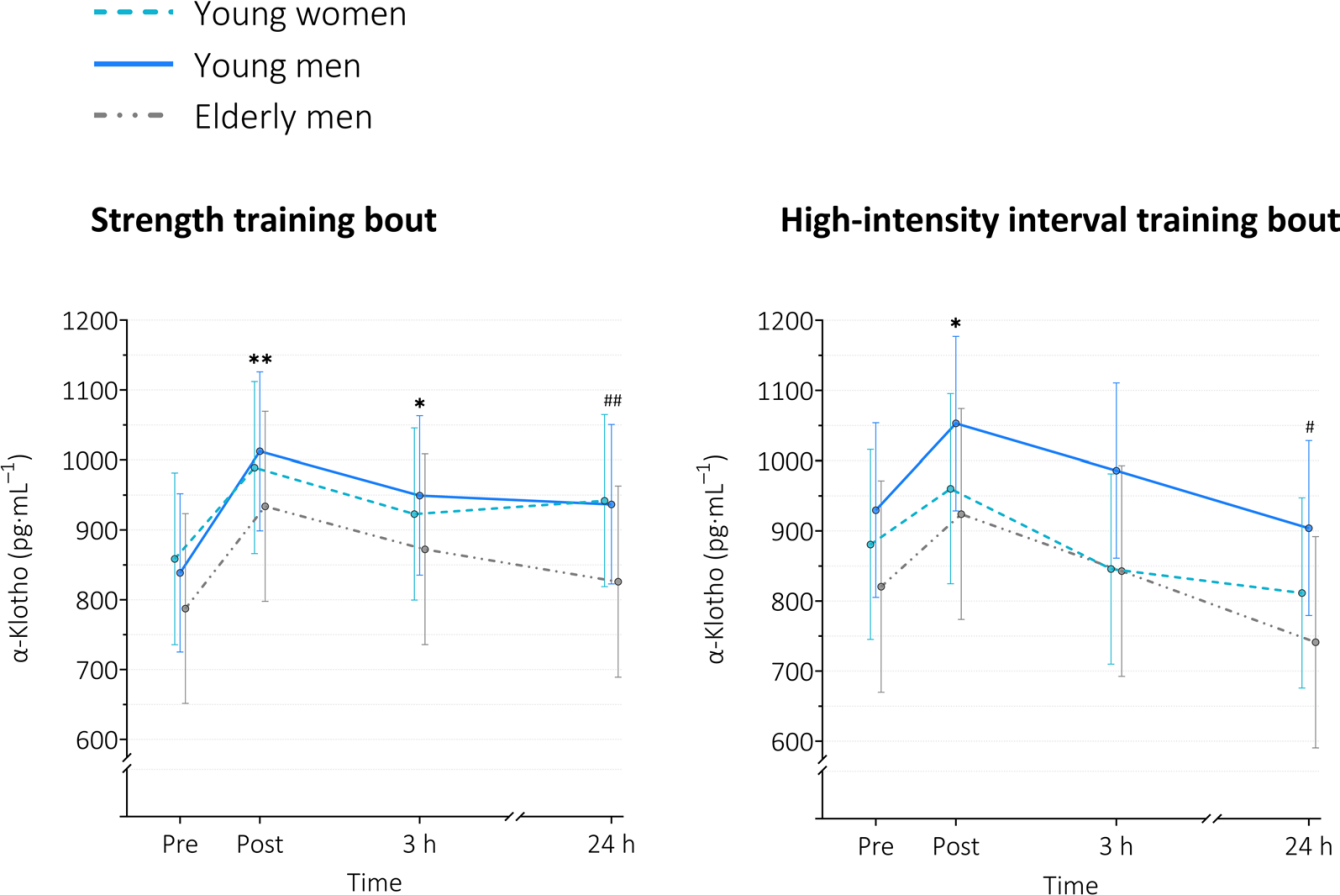
Serum levels of α-Klotho at baseline, immediately, 3 h, and 24 h after exercise. Data are presented as estimated means with error bars representing 95% confidence intervals based on a random intercept model with *time*, *group*, and *time x group* as fixed factors. Young women n = 17 (ST), n = 16 (HIIT); young men n = 20 (ST), n = 19 (HIIT); elderly men n = 14 (ST), n = 13 (HIIT). ST = strength training, HIIT = high-intensity interval training. Pairwise comparisons using Šidák adjustment for multiple testing resulted in the following significant differences (see Table 2 for exact p-values and effect sizes): main effect of time: ** p < 0.001 vs. baseline within all 3 groups; * *p* < 0.01 vs. baseline within all 3 groups; # *p* < 0.05 vs. baseline within young women and elderly men; ## *p* < 0.001 vs. baseline within young men and young women

For ST, a main effect of *time* was observed (*p* < 0.001). At post, serum Klotho levels were increased by 15.2%, 20.7%, and 18.6% in young women, young men, and elderly men, respectively. At 3 h (all groups) and 24 h (young women and young men), levels were increased compared to baseline (Table 2 and Fig. 4). No group effects were found (*p* = 0.618).

**Table 2:** Effect sizes for serum level changes of Klotho, BDNF, and GPLD1 for ST and HIIT

Klotho	ST (n = 51)		HIIT (n = 48)	
Time	MD, [95% CI] in pg $\cdot$ mL^−1^	*p*-value	MD, [95% CI] in pg $\cdot$ mL^−1^	*p*-value
Young women				
Post vs. pre	130.57 [77.77, 183.36]	< 0.001	79.42 [16.73, 142.11]	= 0.006
3 h vs. pre	64.16 [11.37, 116.96]	= 0.009	− 34.99 [− 99.05, 29.07]	= 0.614
24 h vs. pre	83.29 [30.49, 136.08]	< 0.001	− 69.09 [− 133.15, − 5.03]	= 0.028
Young men				
Post vs. pre	173.80 [123.89, 223.70]	< 0.001	123.31 [65.78, 180.83]	< 0.001
3 h vs. pre	110.78 [59.85, 161.70]	< 0.001	56.36 [− 3.37, 116.09]	= 0.075
24 h vs. pre	98.08 [47.16, 149.01]	< 0.001	− 25.65 [− 84,23, 32.92]	= 0.813
Elderly men				
Post vs. pre	146.24 [84.29, 208.20]	< 0.001	103.48 [32.04, 174.92]	= 0.001
3 h vs. pre	84.77 [21.12, 148.41]	= 0.003	22.13 [− 49.31, 93.57]	= 0.958
24 h vs. pre	38.52 [− 27.07, 104.11]	= 0.533	− 79.34 [− 152.73, − 5.95]	= 0.027

BDNF	ST (n = 52)		HIIT (n = 49)	
Time	MD, [95% CI] in pg $\cdot$ mL^−1^	*p*-value	MD, [95% CI] in pg $\cdot$ mL^−1^	*p*-value
Young women				
Post vs. pre	3033.50 [1316.31, 4750.69]	< 0.001	3289.74 [1886.52, 4692.97]	< 0.001
3 h vs. pre	1702.22 [*− *47.78, 3452.22]	= 0.061	* − *222.04 [*− *1688.90, 1244.82]	= 0.999
24 h vs. pre	240.67 [*− *1476.51, 1957.86]	= 0.999	* − *437.63 [*− *1904.49, 1029.23]	= 0.965
Young men				
Post vs. pre	2374.46 [706.69, 4042.24]	= 0.001	3333.35 [1981.89, 4684.81]	< 0.001
3 h vs. pre	559.44 [*− *1108.33, 2227.22]	= 0.939	485.50 [*− *892.46, 1863.47]	= 0.923
24 h vs. pre	* − *176.96 [*− *1844.74, 1490.81]	= 1.00	* − *597.46 [*− *1948.92, 754.00]	= 0.807
Elderly men				
Post vs. pre	2923.66 [800.00, 5047.32]	= 0.002	2742.74 [1138.09, 4347.39]	< 0.001
3 h vs. pre	202.86 [*− *1864.79, 2270.52]	= 1.00	1248.34 [*− *404.76, 2901.44]	= 0.245
24 h vs. pre	* − *2388.52 [*− *4456.17, − 320.86]	= 0.015	117.92 [*− *1585.06, 1820.90]	= 1.00

GPLD1	ST (n = 51)			HIIT (n = 47)		
Time	MD, ratio of means		*p*-value	MD, ratio of means		*p*-value
Young women						
Post vs. pre	1.17	*1.257	= 0.055	0.42	1.061	= 0.955
3 h vs. pre	1.20	1.264	= 0.046	1.17	1.172	= 0.178
24 h vs. pre	1.18	1.259	= 0.044	0.15	1.022	= 1.00
Young men						
Post vs. pre	1.06	1.168	= 0.281	0.69	1.110	= 0.466
3 h vs. pre	0.69	1.110	= 0.777	1.60	1.257	= 0.006
24 h vs. pre	* − *0.11	0.983	= 1.00	1.08	1.173	= 0.084
Elderly men						
Post vs. pre	0.11	1.028	= 1.00	0.22	1.067	= 0.958
3 h vs. pre	0.29	1.071	= 0.984	0.09	1.028	= 1.00
24 h vs. pre	0.04	1.011	= 1.00	* − *0.13	0.960	= 0.099

Data are presented as mean difference (MD) with 95% confidence interval (CI) or ratio of means. *A ratio of means of 1.257 implies that mean serum GPLD1 levels immediately after ST (post) were 1.257 times higher than those at baseline (pre). *BDNF* brain-derived neurotrophic factor, *GPLD1* glycosylphosphatidylinositol specific phospholipase D1, *ST* strength training, *HIIT* high-intensity interval training

For HIIT, a main effect of *time* was observed (*p* < 0.001). At post, serum Klotho levels were increased by 9.0%, 13.3%, and 12.6% in young women, young men, and elderly men, respectively. Serum Klotho levels decreased below baseline levels 24 h after the exercise bout in young women and elderly men (Table 2 and Fig. 4). No group effects were found (*p* = 0.329).

#### BDNF

**Fig. 5:**
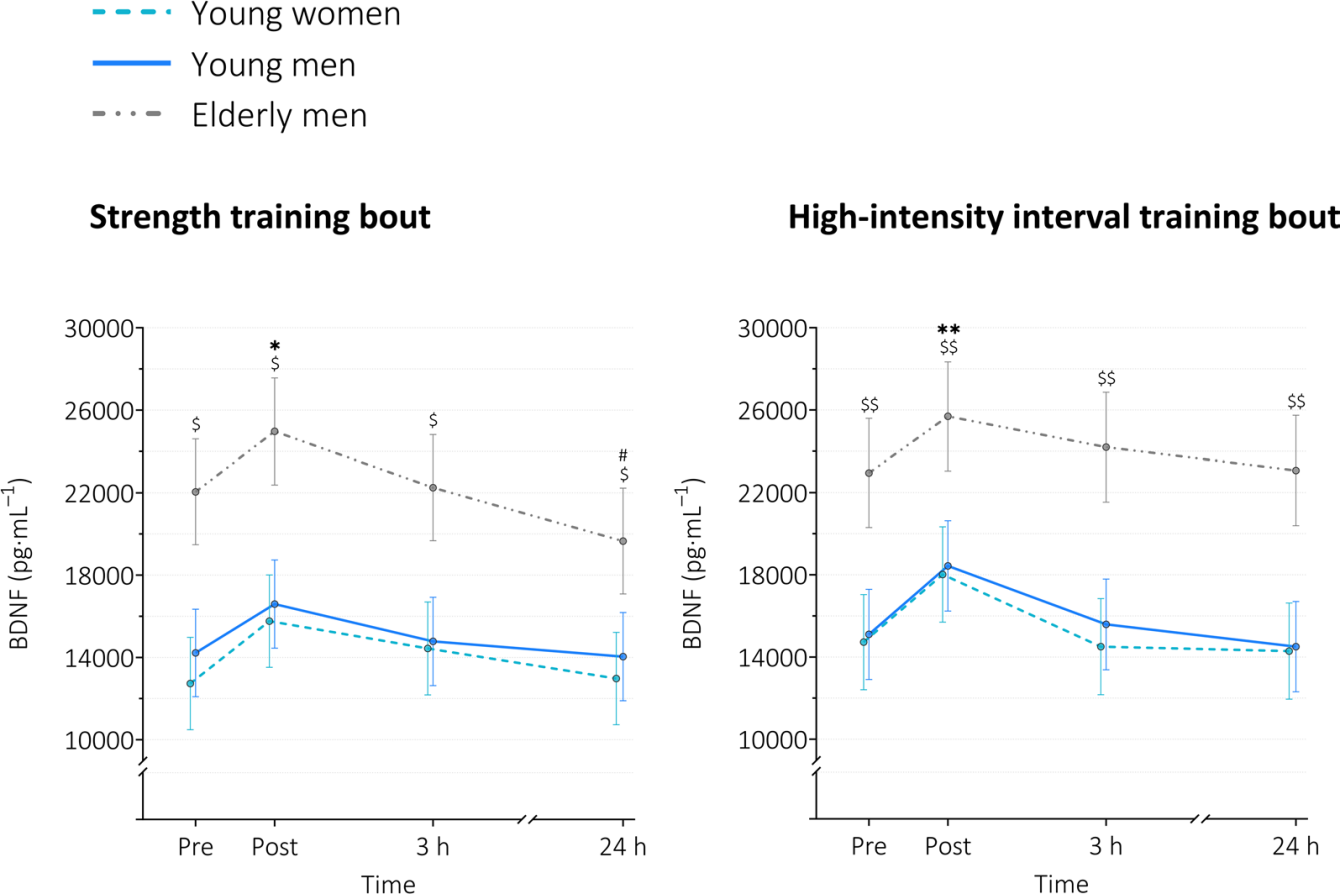
Serum levels of BDNF at baseline, immediately, 3 h, and 24 h after exercise. *BDNF* brain-derived neurotrophic factor. Data are presented as estimated means with error bars representing 95% confidence intervals based on a random intercept model with *time*, *group*, and *time x group* as fixed factors. Young women n = 18 (ST), n = 17 (HIIT); young men n = 20 (ST), n = 19 (HIIT); elderly men n = 14 (ST), n = 13 (HIIT). ST = strength training, HIIT = high-intensity interval training. Pairwise comparisons using Šidák adjustment for multiple testing resulted in the following significant differences (see Table 2 for exact p-values and effect sizes): main effect of time: * *p* < 0.01 vs. baseline within all 3 groups; ** *p* < 0.001 vs. baseline within all 3 groups; # *p* < 0.05 vs. baseline within elderly men; main effect of group: $*p* < 0.01 young men vs. elderly men; $$ *p* < 0.001 young men vs. elderly men

For ST, both main effects of *time* (*p* < 0.001) and *group* (*p* < 0.001) were found to be statistically significant. At post, serum BDNF levels were increased by 23.8%, 16.7%, and 13.3% in young women, young men, and elderly men, respectively. Serum BDNF levels decreased below baseline levels 24 h after the exercise bout in elderly men (Table 2 and Fig. 5). For group effects, BDNF levels were higher in elderly men than in young men at all four time points (pre: MD = 7826.12 pg $\cdot$ mL^−1^, 95% CI [3735.60, 11916.64], *p* < 0.001; post: MD = 8375.32 pg $\cdot$ mL^−1^, 95% CI [4244.91, 12505.72], *p* < 0.001; 3 h: MD = 7469.54 pg $\cdot$ mL^−1^, 95% CI [3359.82, 11579.27], *p* < 0.001; 24 h: MD = 5614.56 pg $\cdot$ mL^−1^, 95% CI [1504.84, 9724.29], *p* = 0.004, Fig. 5).

For HIIT, significant main effects of *time* (*p* < 0.001) and *group* (*p* < 0.001) were observed. At post, serum BDNF levels were increased by 22.3%, 22.1%, and 12% in young women, young men, and elderly men, respectively (Table 2 and Fig. 5). Regarding group effects, BDNF levels were higher in elderly men than in young men at all four time points (pre: MD = 7848.81 pg $\cdot$ mL^−1^, 95% CI [3622.36, 12075.26], *p* < 0.001; post: MD = 7258.20 pg $\cdot$ mL^−1^, 95% CI [3026.04, 11490.36], *p* < 0.001; 3 h: MD = 8611.64 pg $\cdot$ mL^−1^, 95% CI [4359.20, 12864.09], *p* < 0.001; 24 h: MD = 8564.20 pg $\cdot$ mL^−1^, 95% CI [4303.39, 12825.00], *p* < 0.001, Fig. 5)***.***

#### GPLD1

**Fig. 6:**
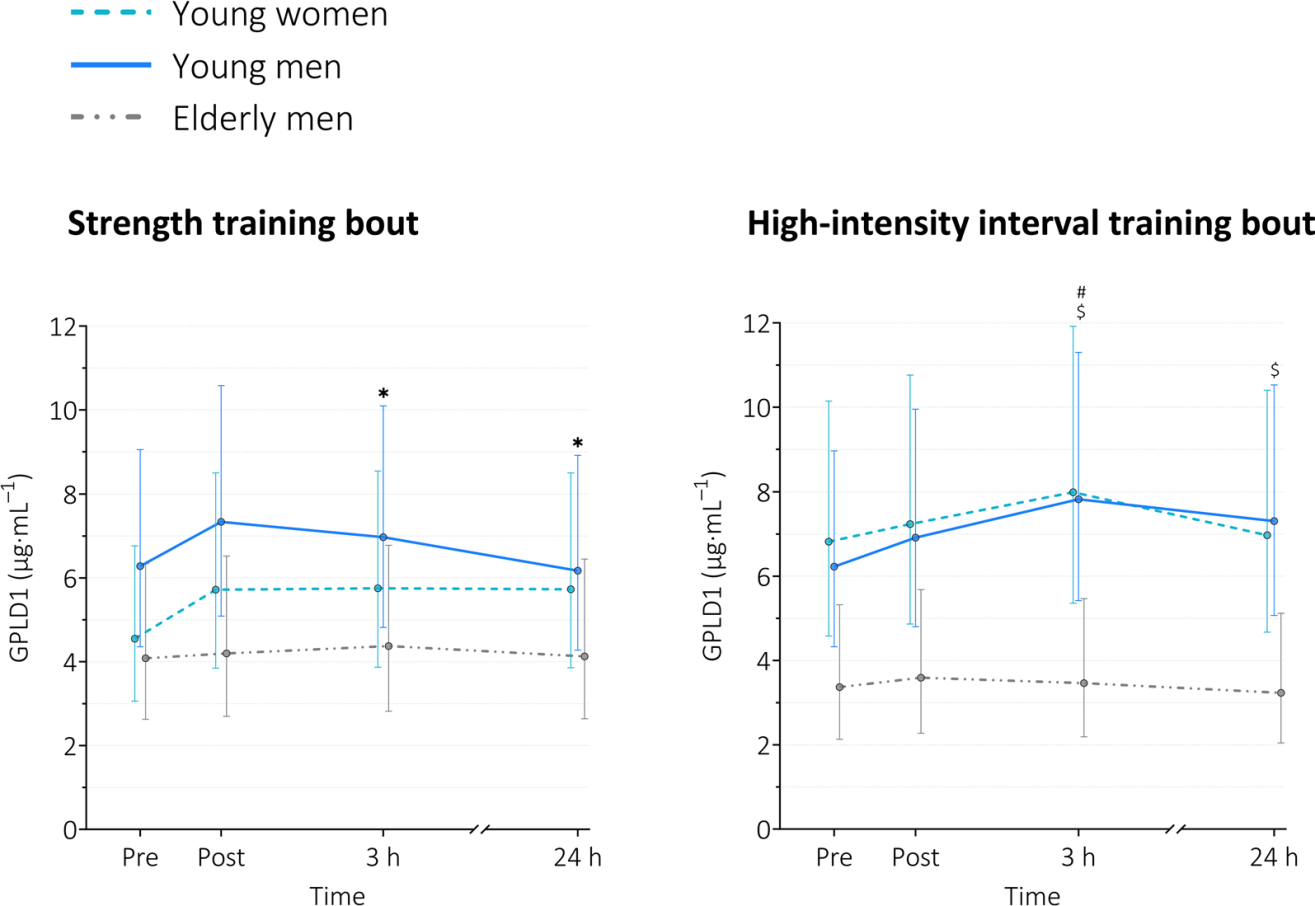
Serum levels of GPLD1 at baseline, immediately, 3 h, and 24 h after exercise. *GPLD1* glycosylphosphatidylinositol specific phospholipase D1. Data are presented as geometric means with error bars representing non-symmetric 95% confidence intervals due to data transformation (natural logarithm). Data are based on a random intercept model with *time*, *group*, and *time x group* as fixed factors. Young women n = 17 (ST), n = 16 (HIIT); young men n = 20 (ST), n = 19 (HIIT); elderly men n = 14 (ST), n = 12 (HIIT). *ST* strength training, *HIIT* high-intensity interval training. Pairwise comparisons using Šidák adjustment for multiple testing resulted in the following significant differences (see Table 2 for exact p-values and effect sizes): main effect of time: * *p* < 0.05 within young women only; # *p* < 0.01 within young men only; main effect of group:$ *p* < 0.05 young men vs. elderly men

For ST, a main effect of *time* (*p* = 0.030) was observed. Serum GPLD1 levels were increased 3 h and 24 h after the exercise bout in young women only (Table 2 and Fig. 6). No group effects were found (*p* = 0.249).

For HIIT, a main effect of *time* (*p* = 0.013) and *group* (*p* = 0.024) was observed. Serum GPLD1 levels were increased 3 h after the exercise bout in young men only (Table 2 and Fig. 6). Regarding group effects, serum GPLD1 levels were higher in young men than in elderly men 3 h and 24 h after the exercise bout (3 h: MD = 4.36 µg $\cdot$ mL^−1^, ratio of means = 2.261, *p* = 0.0221; 24 h: MD = 4.07 µg $\cdot$ mL^−1^, ratio of means = 2.259, *p* = 0.0222, Fig. 6).

### Skeletal muscle gene expression

Overall, statistically significant main effects of *time* were found for all biomarkers. F-statistic values from analysis of overall effects of *time* can be found in Table 3. Variance components estimates for Klotho and GPLD1 (variance, 95% CI, and SD) are presented in Supplementary Table 3***.*** Variance components are omitted for BDNF data.

**Table 3:** F-statistic from analysis of serum levels at baseline and across time, and of gene expression

	Baseline serum levels	Serum levels across time		Skeletal muscle gene expression	
		ST	HIIT	ST	HIIT
Klotho	*F*(numerator df, denominator df) = x				
Time		*F*(3,132.28) = 53.41**	*F*(3,128.07) = 44.96**	*F*(2,29.91) = 6.53*	*F*(2,29.44) = 6.24*
Group	*F*(2,29.77) = 6.19*	*F*(2,48.05) = 0.49	*F*(2,44.99) = 1.14		
Time x group		*F*(6,132.27) = 1.44	*F*(6,128.07) = 1.64		
BDNF	*F*(numerator df, denominator df) = x				
Time		*F*(3,137.72) = 29.53**	*F*(3,127.07) = 47.54**	*F*(2,31.36) = 10.69**	*F*(2,29.74) = 2.92
Group	*F*(2,26.53) = 11.77**	*F*(2,48.09) = 15.57**	*F*(2,45.86) = 15.79**		
Time x group		*F*(6,137.67) = 2.08	*F*(6,127.07) = 1.25		
GPLD1	*F*(numerator df, denominator df) = x				
Time		*F*(3,124.89) = 3.07^#^	*F*(3,116.01) = 3.75^#^	*F*(2,30.31) = 14.85**	*F*(2,30.12) = 9.68**
Group	*F*(2,28.59) = 4.03^#^	*F*(2,48.30) = 1.43	*F*(2,43.83) = 4.09^#^		
Time x group		*F*(6,124.85) = 1.12	*F*(6,116.01) = 1.17		

For baseline serum levels, only F-statistic from analysis of overall *group* effects are reported. For skeletal muscle gene expression, only F-statistic from analysis of overall *time* effects are reported. *ST* strength training, *HIIT* high-intensity interval training, *df* degrees of freedom, *BDNF* brain-derived neurotrophic factor, *GPLD1* glycosylphosphatidylinositol specific phospholipase D1. **p < 0.001, *p < 0.01, ^#^*p* < 0.05

#### Klotho

**Fig. 7:**
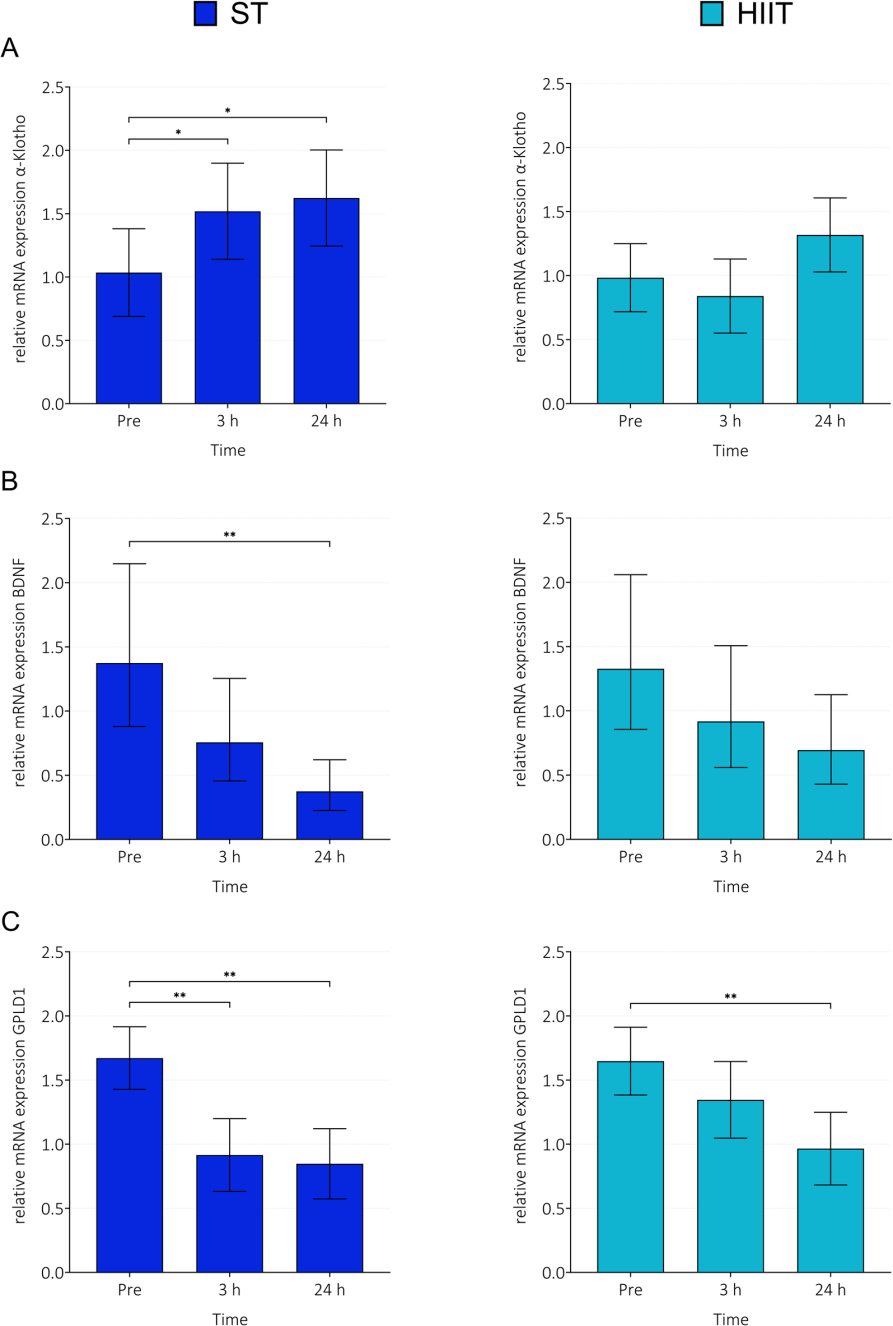
Relative mRNA expression of α-Klotho (**A**), BDNF (**B**) and GPLD1 (**C**) at baseline (pre), 3 h, and 24 h after exercise. *mRNA* messenger RNA, *BDNF* brain-derived neurotrophic factor, *GPLD1* glycosylphosphatidylinositol specific phospholipase D1, *ST* strength training, *HIIT* high-intensity interval training. Data are presented as estimated means and 95% confidence intervals (CIs) based on a random intercept model with *time* as fixed factor (geometric means and non-symmetric 95% CIs for BDNF due to data transformation (natural logarithm)). Klotho n = 21 (ST), n = 22 (HIIT), BDNF n = 21 (ST), n = 22 (HIIT), GPLD1 n = 20 (ST), n = 21 (HIIT). Pairwise comparisons were adjusted for multiple testing (Šidák adjustment). *p < 0.05, **p < 0.001

For ST (Fig. 7A), a main effect of *time* was observed (*p* = 0.004). Relative gene expression was increased at 3 h (MD = 0.48, 95% CI [0.04, 0.93], *p* = 0.029) and 24 h (MD = 0.59, 95% CI [0.15, 1.03], *p* = 0.006).

For HIIT (Fig. 7A), a main effect of *time* was observed (*p* = 0.006). This significant difference is due to an increase from 3 to 24 h. Relative gene expression tended to increase from baseline to 24 h (MD = 0.33, 95% CI [*− *0.002, 0.67], *p* = 0.051).

#### BDNF

For ST (Fig. 7B), a main effect of *time* was observed (*p* < 0.001). Relative gene expression was decreased at 24 h (MD = *− *1.00, ratio of means = 0.272, *p* < 0.001).

For HIIT (Fig. 7B), no main effect of *time* was observed (*p* = 0.070).

#### GPLD1

For ST (Fig. 7C), a main effect of *time* was observed (*p* < 0.001). Relative gene expression was decreased at 3 h (MD = *− *0.76, 95% CI [*− *1.19, − 0.32], *p* < 0.001) and 24 h (MD = *− *0.82, 95% CI [*− *1.25, –0.40], *p* < 0.001).

For HIIT (Fig. 7C), a main effect of *time* was observed (*p* < 0.001). Relative gene expression was decreased at 24 h (MD = *− *0.68, 95% CI [*− *1.07, − 0.29], *p* < 0.001).

## Discussion

The present study investigated acute responses of three cognitive biomarkers to two different exercise modes in young women, young men, and elderly men. Baseline serum levels were also analyzed. The main finding was that acute ST and HIIT transiently increased serum levels of Klotho, BDNF, and GPLD1. Neither sex nor age were found to be determinants of changes in serum levels across time. Age, but not sex, appeared to affect basal Klotho and BDNF levels. While skeletal muscle mRNA expression of Klotho increased, BDNF and GPLD1 expression decreased following exercise.

### Baseline serum levels

Serum Klotho levels did not differ between young men and women, which is in line with Yamazaki et al. [55]. However, we observed that elderly men exhibited lower serum Klotho levels than young men. This finding is consistent with previous studies [55, 56], confirming a negative association between serum Klotho and age. Notably, serum levels of Klotho did not appear to differ between elderly people with high and low physical functioning [56], indicating that aging alone was the determining factor for the decreased Klotho levels at older age. Interestingly, being a master (“veteran”) athlete may, to some extent, protect from the age-related decrease in Klotho levels [57].

Lommatzsch et al. reported that circulating BDNF levels decrease with advancing age [58]. Although this is opposite to significantly higher serum BDNF levels in our group of elderly men compared to the young group, our results are supported by a validation study that reported an increase of 0.33% in serum BDNF levels for each year of age [59]. The contrary findings by Lommatzsch et al. [58] could be due to differing age categories of elderly individuals, i.e., 48–60 years vs. 63–73 years in the present study. Differences in physical fitness level could also influence the results. Namely, the clearance rate of BDNF might be less effective in untrained individuals, causing higher levels of circulating BDNF [60]. This fits to earlier findings of a negative correlation between serum BDNF levels and V̇O_2max_ in a large cohort of healthy men aged 14–76 years [61]. As V̇O_2max_ declines with age [62], this could partly explain the higher BDNF levels observed in elderly men in the present study.

Basal serum GPLD1 levels were not affected by sex, which is in line with Zhang et al. [63]. However, although not significant, age appeared to be an influencing factor, as our results revealed lower GPLD1 levels in elderly men than in young men. This finding is supported by a reported inverse relationship of serum GPLD1 levels with age [64]. Mechanisms underlying reduced serum GPLD1 levels observed with age are unclear, but explanations include decreased or deficient synthesis of GPLD1 or reduced liver volume at higher age [64].

### Serum level changes across time

Limited data are available regarding acute responses of circulating Klotho levels to exercise. In terms of aerobic training, Tan et al. reported an approximately 25% increase in serum Klotho levels in middle-aged adults after high-intensity exercise [14]. This finding is in line with our study, though we observed a smaller change across groups (11.6%). Additionally, Tan et al. reported a decrease in Klotho levels close to baseline values 30–240 min after exercise [14]. We observed a similar drop 3 h after HIIT, with levels further decreasing during the following 21 h. Whereas Klotho levels in the present group of young men increased by 20.8% after ST, Morishima and Ochi reported nearly doubled serum Klotho levels immediately after one acute ST session [15]. This discrepancy could be explained by lower basal Klotho levels in the abovementioned study (approximately 200 pg $\cdot$ mL^−1^) than those in the present study (838 pg $\cdot$ mL^−1^), which in turn could be due to the difference in training status. Moreover, plasma volume changes can occur after ST [50], and the lack of adjusting for such alterations could also explain the diverging findings. Noteworthy, in contrast to these results, Iturriaga et al. observed decreased Klotho levels immediately after ST in strength-trained young men, followed by a peak first 24–48 h after exercise [65]. We also found that Klotho levels were increased 3 h and 24 h after exercise compared to baseline levels (young group only), although the levels were lower than immediately after. These conflicting findings are hard to resolve, but differences may be attributed to analytical procedures and different exercise characteristics.

Neither sex nor age appeared to affect the acute response of serum Klotho levels to exercise. In particular, baseline serum Klotho levels differed between young and elderly men, yet a similar increase occurred from pre to post in both groups. This is partly in line with Avin et al. who also observed an increase in circulating Klotho levels in overweight-obese young and older women after one bout of aerobic light- to moderate-intensity exercise [66]. The increase in the elderly group was approximately half that of the young group, but it was not statistically significant [66]. Thus, a novel finding in our study is that both one bout of ST and HIIT promoted a similar change in serum Klotho levels, irrespective of age. The present results support the notion that circulating Klotho levels transiently increase after acute exercise, although the origin of this response is unclear.

Acute aerobic exercise has been shown to induce increases in circulating BDNF levels in healthy adults [23, 24, 67]. Our study expands these findings by showing that such an increase is independent of sex and age. However, less information is available regarding acute effects of ST. A meta-analysis on the relationship between circulating BDNF and exercise reported that only 8 of 55 included studies examined ST [68]. Furthermore, only three of these ST trials showed a significant effect on circulating BDNF levels. Moreover, a recent systematic review reported inconsistent findings regarding changes in circulating BDNF levels after acute heavy ST [27]. Our findings demonstrated that one bout of ST induced a significant and similar increase of serum BDNF levels in individuals across different ages and sex immediately after exercise. Interestingly, plasma BDNF levels have been found to remain elevated up to 1 h after heavy ST [69, 70], and we cannot rule out that serum BDNF levels kept increasing after post in our study, before decreasing to baseline levels 3–24 h after ST. However, another study has demonstrated that BDNF levels decrease below baseline levels within 1 h after ST [71]. Although comparable studies are few and findings are inconsistent, our data demonstrate that one bout of either ST or HIIT induced similar increases in serum BDNF levels, across groups of different age and sex.

Recently, Horowitz et al. proposed a connection between GPLD1 and neurogenesis/cognition in mice [16]. Since then, several reviews [1, 72, 73] reported on the potential effects of exercise-induced increases in GPLD1 levels on brain function. A novelty of our study is the investigation of acute effects of exercise on serum GPLD1 levels in healthy human individuals. Our findings demonstrate that ST and HIIT led to a transient increase in GPLD1 levels, with a peak 3 h after exercise. The finding that Klotho and BDNF levels peaked immediately after exercise, along with a larger relative change, might indicate that GPLD1 is released more slowly, for example via involvement of secondary mechanisms, and/or is less responsive to exercise. Additionally, the increase was only significant in the young group, which could imply that serum GPLD1 levels in elderly are less modifiable by acute exercise. Interestingly, in a cross-sectional approach, Horowitz et al. found higher levels of plasma GPLD1 in physically active older adults than in sedentary adults of similar age [16]. Therefore, chronic exercise might be necessary to upregulate GPLD1 activity in the elderly.

### Skeletal muscle gene expression

Klotho is mainly expressed in the kidneys, brain, and pituitary gland, with lower expression in skeletal muscle [74]. There is a lack of studies exploring skeletal muscle expression of Klotho following acute exercise in humans. In the present study, the relative mRNA expression of Klotho increased within 24 h following both exercise modes (trend for HIIT). One study in mice found reduced Klotho mRNA expression in skeletal muscle within 12 h after aerobic exercise, followed by an increase at 72 h [75]. The same study also reported increased Klotho protein expression 24 h following exercise. Although the increased Klotho mRNA expression we observed is somewhat unlike the aforementioned findings in mice [75], factors such as species-related differences, exercise characteristics and timing of biopsies may contribute to deviating results.

While the brain is known as the main site of BDNF expression [76], other tissues such as skeletal muscle and endothelial cells also express BDNF [30, 31, 77]. Matthews et al. reported that BDNF expression in skeletal muscle was upregulated first several hours after exercise [31], excluding any contribution to immediate BDNF release into the circulation. The authors suggested that BDNF appears to function in a para- and autocrine manner instead [31]. In the present study, BDNF mRNA expression was mainly reduced, although only significantly after ST. A previous study in humans reported unaltered skeletal muscle mRNA expression of BDNF, but increased protein expression in muscle within 90 min following knee-extension exercise [28]. De Assis et al. observed decreased BDNF mRNA expression in skeletal muscle immediately after all-out running exercise [78], which is somewhat similar to our results following ST, although the reduction was most evident after 24 h. Whether skeletal muscle BDNF release contributes to increased circulating BDNF levels following exercise remains inconclusive.

While GPLD1 is mainly synthesized in the liver [79], it has also been detected in skeletal muscle of mice [16]. Interestingly, Horowitz et al. showed that mice subjected to exercise exhibited increased GPLD1 mRNA expression in the liver, without any changes in skeletal muscle and brain [16], suggesting that the liver is the main site of exercise-induced GPLD1 release. Our results showed that GPLD1 mRNA is expressed in skeletal muscle at rest but decreases within 24 h after ST and HIIT. However, these findings should be interpreted with caution since muscle mRNA response may be influenced by several factors (e.g., species-specific characteristics, exercise type and timing of biopsies). It should also be reminded that biopsy-derived mRNA results following exercise represent a snapshot in time, which does not necessarily correspond with subsequent protein expression [80]. It needs to be clarified whether acute exercise induces GPLD1 release from muscle or mainly stimulates release of available GPLD1 from other sources.

### Implications and future directions

Recent studies have highlighted several relevant biomarkers related to neurocognition [7, 72, 81]. The potential for use of key proteins involved in the interplay between brain health and exercise as diagnostic markers and treatment-evaluation tools in psychiatry is emerging [82]. Importantly, as our study demonstrated, acute exercise effectively increased serum levels of Klotho, BDNF, and GPLD1, which have been associated with healthy brain function and neurocognition [11, 16, 83]. The serum responses of Klotho and GPLD1 are particularly interesting, because few studies have explored their potential role in exercise-mediated benefits for brain health. Potentially, the accumulated effect of repeated bouts of acute exercise (i.e., chronic exercise) could elicit long-term changes by altering resting levels, ultimately triggering physiological or structural adaptations that could benefit brain function and cognition [10, 81]. Since exercise-induced increases in BDNF levels appear to benefit brain function and cognition [84], it is of particular interest that serum Klotho levels increased to a similar extent as serum BDNF levels. This indicates that Klotho and BDNF could be comparably stimulated by exercise, which might translate to beneficial effects on brain function. Clearly, more studies are needed to investigate this claim and the role of Klotho in the exercise-cognition interplay. Furthermore, evaluating potential associations between biomarker changes and cognitive performance is important to develop and advance therapeutic approaches directly targeting these proteins. In the long run, this could provide new treatment options for mental and cognitive disorders and eventually decrease their disease burden.

### Strength and limitations

One strength of the present study is its within-subjects crossover design, which increases statistical power by removing bias due to inter-individual biological variation [85]. Another strength is the relatively high number of participants across sex and age, along with the inclusion of two training modes. Furthermore, we applied a plasma volume correction using changes in calcium levels as a reference, since potential changes in plasma volume after exercise could affect biomarker levels [50–52]. Moreover, blood samples were taken in a standardized manner with regard to fasting state to minimize pre-analytical variability, and at fixed time points during the day to avoid diurnal variations. In addition, qualified staff supervised all exercise sessions and tests to ensure correct execution. As for limitations, including more time points for post exercise blood measurements would have been an advantage, especially between post and 3 h after exercise. Hence, we might have missed the exact point of peak serum levels for the different biomarkers. However, four time points were considered sufficient in terms of participant strain and feasibility. At last, two limitations emerge from this study being a secondary analysis of previously published data material [44]. Firstly, the lack of elderly women to evaluate the effect of sex with age. Secondly, we did not test cognition before and after acute ST and HIIT as cognitive assessment was not a planned part of the original study. Regarding muscle gene expression analyses, we could not evaluate sex or age differences due to the relatively low sample size in the subgroups of biopsy volunteers. Also, including protein expression analyses in the biopsies would have been beneficial to interpret the muscle-specific response from exercise.

## Conclusion

Neurocognitive function is a vital part of overall health and exercise has emerged as a promising measure to maintain and improve brain health. It is therefore pertinent to explore relevant neurocognitive proteins and their response to exercise. The current findings demonstrated that ST and HIIT acutely increased serum levels of Klotho, BDNF, and GPLD1. Notably, although age affected baseline levels, it was not a determining factor for changes across time, as all groups showed similar time course kinetics after exercise. The altered skeletal muscle mRNA expression of Klotho, BDNF, and GPLD1 following both exercise modes indicates that skeletal muscle might play a role in exercise-induced elevations of these proteins. Ultimately, both transient increases and elevated resting levels of neuroprotective proteins following exercise may have implications as a non-pharmacological effort to manage neurocognitive decline in ageing or disease. 

## Supplementary Information

Supplementary material 1.

## Data Availability

The datasets used and analyzed during the current study are available from the corresponding author on reasonable request.

## Abbreviations

BDNF: Brain-derived neurotrophic factor
GPLD1: Glycosylphosphatidylinositol-specific phospholipase D1
mRNA: Messenger RNA
ST: Strength training
HIIT: High-intensity interval training
BMI: Body mass index
1RM: One repetition maximum
V̇O_2max_: Maximal oxygen uptake
HR_max_: Maximal heart rate
ELISA: Enzyme-linked immunosorbent assay
PV: Plasma volume
[Ca]: Calcium concentration
cDNA: Complementary DNA
PCR: Polymerase chain reaction
GAPDH: Glyceraldehyde-3-phosphate dehydrogenase
ANOVA: Analysis of variance
MD: Mean difference
CI: Confidence interval
DF: Degrees of freedom
