# Supplementary material for: Acute effects of a single bout of high-intensity strength and endurance exercise on cognitive biomarkers in young adults and elderly men: a within-subjects crossover study
Source: J Transl Med. 2025 Jun 19;23:685. doi: 10.1186/s12967-025-06685-y (PMC12177964; doi:10.1186/s12967-025-06685-y)
Supplement: Supplementary file 1 — Supplementary material 1. [file 12967_2025_6685_MOESM1_ESM.docx]

## Supplementary material

Missing data: Fifty-three individuals were included in the study, i.e., 424 observations, or more specifically, 212 observations in each training mode. Our data displays missing counts (i.e., observations) for the dependent variable only; for Klotho, BDNF, and GPLD1 the missing counts were 39 (9.2%), 38 (9%), and 61 (14.4%) respectively. Data are missing randomly across different time points and all groups. Reasons for missing values are absence of participants/withdrawal, and difficulties with blood sampling or serum level analysis. (Restricted) maximum likelihood estimation can handle missing data and yield unbiased estimates when missing (completely) at random.

Centering: No centering procedures were applied because only categorically coded predictors were used. *Time* was coded such that the pre-measurement (t_1_) served as the reference category. *Group* was coded such that *young men* served as the reference category. Thus, estimates of intercepts for fixed effects were interpreted as serum levels at baseline for young men.

**Supplementary Table 1**: Intraclass correlation coefficients for all biomarkers for ST and HIIT based on the null model

|  | ST | HIIT |
| --- | --- | --- |
| Klotho | 0.89 | 0.88 |
| BDNF | 0.73 | 0.81 |
| GPLD1* | 0.91 | 0.94 |

*based on log-transformed data

ST = strength training, HIIT = high intensity interval training

BDNF = brain-derived neurotrophic factor, GPLD1 = glycosylphosphatidylinositol-specific phospholipase D1.

**Supplementary Table 2:** Variance components for serum level analyses of α-Klotho and BDNF

| **Klotho** |  | **Variance, [95% CI]** | **SD** in pg$\cdot$mL^−1^ |
| --- | --- | --- | --- |
| **ST** |  |  |  |
| Between subjects | 60502.04 | [40298.60, 90834.35] | 245.97 |
| Within subjects | 3320.83 | [2609.10, 4226.72] | 57.63 |
| **HIIT** |  |  |  |
| Between subjects | 68286.53 | [44855.71, 103956.66] | 261.32 |
| Within subjects | 4402.16 | [3445.59, 5624.28] | 66.35 |

| **BDNF** |  | **Variance, [95% CI]** | **SD** in pg$\cdot$mL^−1^ |
| --- | --- | --- | --- |
| **ST** |  |  |  |
| Between subjects | 18949963.63 | [12424458.26, 28902758.91] | 4353.16 |
| Within subjects | 3723888.02 | [2938922.63, 4718512.09] | 1929.74 |
| **HIIT** |  |  |  |
| Between subjects | 20351654.92 | [13340940.03, 31046527.24] | 4511.28 |
| Within subjects | 2342964.68 | [1831828.97, 2996722.73] | 1530.67 |

Data are presented as variance with 95% confidence intervals (CIs), and standard deviation (SD). BDNF = brain-derived neurotrophic factor, ST = strength training, HIIT = high-intensity interval training.

**Supplementary Table 3:** Variance components for mRNA expression analyses of α-Klotho and GPLD1

| **Klotho** |  | **Variance, [95% CI]** | **SD** |
| --- | --- | --- | --- |
| **ST** |  |  |  |
| Between subjects | 0.35 | [0.16, 0.80] | 0.59 |
| Within subjects | 0.24 | [0.14, 0.40] | 0.49 |
| **HIIT** |  |  |  |
| Between subjects | 0.22 | [0.10, 0.49] | 0.47 |
| Within subjects | 0.13 | [0.08, 0.23] | 0.36 |

| **GPLD1** |  | **Variance, [95% CI]** | **SD** |
| --- | --- | --- | --- |
| **ST** |  |  |  |
| Between subjects | 0.05 | [0.01, 0.44] | 0.22 |
| Within subjects | 0.23 | [0.14, 0.40] | 0.48 |
| **HIIT** |  |  |  |
| Between subjects | 0.14 | [0.05, 0.39] | 0.37 |
| Within subjects | 0.19 | [0.11, 0.33] | 0.44 |

Data are presented as variance with 95% confidence intervals (CIs), and standard deviation (SD). mRNA = messenger RNA, GPLD1 = glycosylphosphatidylinositol specific phospholipase D1, ST = strength training, HIIT = high-intensity interval training.
